# Sympathovagal Imbalance in Prehypertensive Offspring of Two Parents versus One Parent Hypertensive

**DOI:** 10.4061/2011/263170

**Published:** 2011-10-23

**Authors:** G. K. Pal, Pravati Pal, Nivedita Nanda, V. Lalitha, T. K. Dutta, C. Adithan

**Affiliations:** ^1^Department of Physiology, Jawaharlal Institute of Post-Graduate Medical Education and Research (JIPMER), Puducherry 605 006, India; ^2^Department of Biochemistry, Pondicherry Institute of Medical Sciences (PIMS), Puducherry 605 014, India; ^3^Department of Medicine, Jawaharlal Institute of Post-Graduate Medical Education and Research (JIPMER), Puducherry 605 006, India; ^4^Department of Pharmacology, Jawaharlal Institute of Post-Graduate Medical Education and Research (JIPMER), Puducherry 605 006, India

## Abstract

*Objective*. Though prehypertension has strong familial predisposition, difference in pathophysiological mechanisms in its genesis in offspring of both parents and single parent hypertensive have not been elucidated. *Methods*. Body mass index (BMI), waist-hip ratio (WHR), basal heart rate (BHR), blood pressure (BP), HR and BP response to standing, deep breathing difference, BP response to handgrip and spectral indices of heart rate variability (HRV) were analyzed in normotensive offspring of two parents hypertensive (Group I), normotensive offspring of one parent hypertensive (Group II), prehypertensive offspring of two parents hypertensive (Group III) and prehypertensive offspring of one parent hypertensive (Group IV). *Results*. Sympathovagal imbalance (SVI) in prehypertensive offspring was observed due to increased sympathetic and decreased vagal activity. In group III, SVI was more prominent with greater contribution by vagal withdrawal. LF-HF ratio, the marker of SVI was correlated more with diastolic pressure, 30 : 15 ratio and E : I ratio in prehypertensives and the degree of correlation was more in group III prehypertensives. *Conclusion*. Vagal withdrawal plays a critical role in development of SVI in prehypertensive offspring of hypertensive parents. The intensity of SVI was more in offspring of two parents hypertensive compared to single parent hypertensive.

## 1. Introduction

Hypertension runs in families, and parental history of hypertension increases the risk of developing hypertension, especially if both the parents are hypertensives [1]. Autonomic abnormality in the form of increased sympathetic tone has been demonstrated in young normotensive offspring of hypertensive parents [2]. Also, it was observed that youngsters with a parental history of hypertension showed an enhanced reactivity of total peripheral resistance during static exercise without higher BP response as the physiological increase in stroke volume was blunted in these subjects [3]. Recently, it has been reported that cardiovascular autonomic responses to whole body isotonic exercise in normotensive healthy young adult males with parental history of hypertension show signs of sympathetic overactivity [4]. However, till date, no work has been conducted to understand the nature of autonomic imbalance that facilitates the normotensive offspring of hypertensive parents to enter into the stage of prehypertension or hypertension. A recent report from our laboratory suggests that increased sympathetic and decreased parasympathetic activities in young adults alters sympathovagal balance, which could be the major mechanism in the causation of prehypertension [5]. 

Prehypertension has recently been documented to be associated with increased rate of adverse cardiovascular events [6–8]. Though several studies have assessed sympathovagal imbalance in hypertensive patients [9–12], and sustained sympathetic overactivity has been reported as among the primary mechanisms for genesis of essential hypertension [13–18], there is paucity of data on the nature of change in autonomic balance that slowly changes the normotensive state to develop into the state of prehypertension. Though hypertension is common in middle aged and elderly population [19], prehypertension is relatively more common in young adults, especially in those who have family history of hypertension [20]. Recently, we have observed decrease in vagal tone contributes profoundly to the development of prehypertension in the offspring of hypertensive parents (unpublished materials). However, till date, no study has been conducted to analyze the magnitude and pattern of sympathovagal imbalance in prehypertensive offspring of both-parents hypertensive and single-parent hypertensive to understand the pathophysiological basis and cardiovascular risks in these two genetically predisposed categories of young population. 

Spectral analysis of heart rate variability (HRV) has recently been used as a sensitive tool for assessment of autonomic dysfunctions in various clinical disorders [21]. Therefore, in the present study, we have analyzed the spectral indices of HRV in these two categories of siblings of hypertensive parents to understand how autonomic imbalance results in prehypertension in them and if this knowledge can be utilized to evolve the strategies for prevention of prehypertension. 

## 2. Methods

### 2.1. Subjects

After obtaining the approval of Research Council and Institutional Ethics Committee, of Jawaharlal Institute of Postgraduate Medical Education and Research (JIPMER), Puducherry, India, out of 420 students from first year MBBS, M. Sc. (Medical Biochemistry), B. Sc. (Medical Laboratory Technology) and various MD courses of JIPMER of 2008, 2009, and 2010 batches, 172 students with parental history of hypertension were recruited for the study. They were classified into following four groups based on their parental history of hypertension and the level of systolic and diastolic blood pressure as per JNC-7 classification [22].

1. Group I (normotensive offspring of both-parents hypertensive, *n* = 45): offspring of two-parents hypertensive, having systolic BP 100–119 mm Hg and diastolic BP 60–79 mmHg.
2. Group II (normotensive offspring of one-parent hypertensive, *n* = 68): offspring of one-parent hypertensive, having systolic BP 100–119 mm Hg and diastolic BP 60–79 mmHg.
3. Group III (prehypertensive offspring of both-parents hypertensive, *n* = 32): offspring of two-parents hypertensive, having systolic BP 120–139 mm Hg and diastolic BP 80–89 mmHg.
4. Group IV (prehypertensives offspring of one-parent hypertensive, *n* = 20): offspring of one-parent hypertensive, having systolic BP 120–139 mm Hg and diastolic BP 80–89 mmHg.

Subjects regularly practicing athletic activities, with history of smoking, receiving any medication, known to have diabetes, hypertension, kidney disease, or any endocrinal disorder were excluded from the study. 

### 2.2. Laboratory Conditions and Recording of Data

Subjects were asked to report to autonomic function testing (AFT) laboratory of Physiology Department at about 9 AM following a light breakfast, without tea or coffee. After obtaining the informed consent, their age, height, body weight, body mass index (BMI), and waist-hip ratio (WHR) were reordered. The temperature of AFT laboratory was maintained at 25°C for all the recordings. BP of all the subjects were recorded in autonomic functions testing (AFT) laboratory, following the standard procedure [23]. Omron (SEM 1 Model), the automatic blood pressure monitor (Omron Healthcare Co. Ltd, Kyoto, Japan) was used for BP recording. The cuff size of Omron was 121 mm  (width) × 446 mm  (length). The length of the cuff tube was 600 mm. For BP recording, the subject was asked to seat upright with back straight on a wooden armed chair keeping one forearm on a wooden table kept in front and close to the subject. The height of the table was such that the middle of the arm placed on the table approximately coincided with the level of the heart. The subject was asked to keep the other forearm on the side hand rest of the chair. The BP cuff was tied just tight (neither too tight nor loose) on the arm approximately 2 cm above the cubital fossa. It was ensured that the BP cuff was at the level of the heart. After five-minute rest in the same sitting posture, the “Start” button of Omron was pressed that automatically inflated and deflated the cuff and SBP, DBP and basal heart rate (BHR) were noted from the display screen of the equipment. For each subject, SBP, DBP, and BHR were recorded in each arm two times at an interval of five minutes between the recordings, and for each parameter, the mean of the four recordings was considered. 

### 2.3. Recording of HRV Data

After 15 minutes of supine rest on a couch in AFT lab, ECG was recorded for 5 minutes for short-term HRV analysis following the standard procedure as described earlier [24]. For recording of HRV, recommendation of the Task Force on HRV was followed [25]. For the purpose, ECG electrodes were connected and Lead II ECG was acquired at a rate of 1000 samples/second during supine rest using BIOPAC MP 100 data acquisition system (BIOPAC Inc., USA). The data was transferred from BIOPAC to a windows-based PC with Acknowledge software version 3.8.2. Ectopics and artefacts were removed from the recorded ECG. HRV analysis was done using the HRV analysis software version 1.1 (Bio-signal Analysis group, Finland) and fast Fourier transform (FFT) algorithm. Frequency domain such as total power (TP), normalized LF power (LFnu), normalized HF power (HFnu), LF-HF ratio, and time-domain indices such as mean heart rate (mean RR), square root of the mean squared differences of successive normal to normal intervals (RMSSD), the number of interval differences of successive NN intervals greater than 50 ms (NN50) and the proportion derived by dividing NN50 by the total number of NN intervals (pNN50) were calculated. 

### 2.4. Other Autonomic Function Tests

Other autonomic function tests (AFTs) were performed following the standard procedures [26].

#### 2.4.1. Lying to Standing Test

In this test, heart rate (HR) and blood pressure (BP) response to standing was assessed. The BP and ECG were recorded in supine position. The subject was instructed to attain standing posture in 3 seconds. The ECG was continuously recorded during the procedure. The BP was recorded every 40 seconds by automatic BP monitor (Omron, SEM-1) till 5th min. 30 : 15 ratio (ratio of maximum RR interval at 30th beat to minimum RR interval at 15th beat following standing), ΔHR_max_ (maximum rise in HR from supine HR level following standing), ΔHR_2min_ (maintained increase in HR at 2 minutes, from supine HR level following standing), ΔSBP_max_ (maximum rise in SBP from supine SBP following standing), ΔSBP_2min_ (maintained increase in SBP after 2 minutes from supine SBP level following standing), ΔDBP_max_ (maximum rise in DBP from supine DBP following standing), and ΔDBP_2min_ (maintained increase in DBP after 2 minutes from supine DBP level following standing) were calculated.

#### 2.4.2. Deep Breathing Test

The subject in sitting posture, the heart rate and respiration monitoring was done from ECG recording and stethographic respiratory tracings recorded on the polygraph (Nihon-Kohden, UK). A baseline recording of ECG and respiration was taken for 30 seconds. The subject was asked to take slow and deep inspiration followed by slow and deep expiration such that each breathing cycle lasted for 10 seconds, consisting of six breathing cycles per minute. E : I ratio (ratio of average RR interval during expiration to average RR interval during inspiration in six cycles of deep breathing) was calculated from ECG tracing. 

#### 2.4.3. Isometric Handgrip Test

The baseline BP was recorded. The subject was asked to press handgrip dynamometer at 30% of maximum voluntary contraction for 2 minutes. The BP was recorded at 1st minute and 2nd minute of contraction. ΔDBP_IHG_ (maximum rise in diastolic BP above baseline) was noted.

Out of 172 students with history of parental hypertension, seven students reported as known hypertensives, receiving antihypertensive drugs. Therefore, their recordings were not considered for statistical analysis. These seven hypertensive students had both parents hypertensive.

### 2.5. Statistical Analysis of Data

SPSS version 13 (SPSS Inc., Chicago, Ill, USA) and GraphPad InStat softwares (GraphPad Software Inc., San Diego, Calif, USA) were used for statistical analysis. All the data were presented as mean ± SD. Statistical analysis of data was done by one-way ANOVA, and post hoc by Tukey-Krammer test. The *P* values less than 0.05 was considered statistically significant. The association between LF-HF ratio with BMI, WHR, BHR, blood pressure, 30 : 15 ratio, and E : I ratio was assessed by Pearson correlation analysis. 

## 3. Results

In the present study, the incidence of offspring born to parents with hypertension was 40.95% (172/420) and among them the incidence of prehypertension was 30.23% (52/172). 

There was no significant difference in age among all the four groups (Table 1). Body weight, BMI, and WHR of subjects of group III (prehypertensive offspring of both parents hypertensive) was significantly more than group I (normotensive offspring of both parents hypertensive) (*P* < 0.05) and II (normotensive offspring of one parents hypertensive) (*P* < 0.05 for body weight and *P* < 0.01 for BMI and WHR). Basal heart rate (BHR) of Groups III significantly more compared to that of group I (*P* < 0.01) and group II (*P* < 0.001), respectively. BHR of group IV was significantly more (*P* < 0.05) compared to that of Group II. The SBP and DBP of Groups III and IV were significantly high compared to that of Group I (*P* < 0.001) and Group II (*P* < 0.001) (Table 1). 

Total power (TP) of HRV spectrum of Group III was significantly less (*P* < 0.001) compared to that of groups I and II and TP of Group IV was significantly lower than the TP of group I (*P* < 0.05) and Group II (*P* < 0.01) (Table 2). Though LF_nu_ of Group III and IV was considerably more than that of Group I and II, respectively, the differences were not statistically significant. The HF_nu_ of Group III was significantly less compared to the HF_nu_ of Group I (*P* < 0.05) and group II (*P* < 0.01). The LF-HF ratio of Group III was significantly more (*P* < 0.001) compared to that of Groups I and II and LF-HF ratio of Group IV was significantly more compared to Group I (*P* < 0.01) and Group II (*P* < 0.001). Also, LF-HF ratio of Group III was significantly more (*P* < 0.05) compared to that of Group IV. 

The decrease in mean-RR in Group III was significant (*P* < 0.05) compared to that of Group II (Table 3). The RMSSD of Group III was significantly less (*P* < 0.05) compared to that of the values Group I (*P* < 0.05) and Group II (*P* < 0.01).

30 : 15 ratio of Group III was significantly more than that of group I (*P* < 0.05) and group II (*P* < 0.01) and of Group IV was more than that of Group II (*P* < 0.05) (Table 4). ΔHR_max_ of Group III was significantly more compared to that of the values of Groups I and II (*P* < 0.001). ΔHR_max_ of Group IV was significantly more than that of Groups I (*P* < 0.05) and II (*P* < 0.001) and less than that of Group III (*P* < 0.01). ΔHR_2min_ of Group III was significantly more compared to that of the values of Groups I and II (*P* < 0.001). Δ HR_2min_ of Group IV was significantly more than that of Group II (*P* < 0.01) and less than that of Group III (*P* < 0.01). 

ΔSBP_max_ of Group III was significantly more compared to that of the values of Groups I and II (*P* < 0.001) (Table 4). ΔSBP_max_ of Group IV was significantly more than that of group II (*P* < 0.01) and less than that of Group III (*P* < 0.05). ΔSBP_2min_ of Group II was significantly less compared to that of the value of Groups I (*P* < 0.001). ΔSBP_2min_ of Group III was significantly more compared to that of the values of Groups I and II (*P* < 0.001). ΔSBP_2min_ of Group IV was significantly more than that of Group II and less than that of Group III (*P* < 0.001). 

ΔDBP_max_ of Group III was significantly more compared to that of the values of Groups I and II (*P* < 0.001) (Table 4). ΔDBP_max_ of Group IV was significantly more than that of Group II (*P* < 0.01). ΔDBP_2min_ of Group III was significantly more compared to that of the values of Groups I and II (*P* < 0.001). ΔDBP_2min_ of Group IV was significantly less than that of Group III (*P* < 0.05). 

E : I ratio of Group III (*P* < 0.001) was significantly less compared to that of Groups I (*P* < 0.05) and II (*P* < 0.001), and of Group IV was significantly less compared to group II (*P* < 0.05) (Table 4). ΔDBP_IHG_ of Groups III and IV was significantly more compared to that the values of Groups I and II (*P* < 0.001), and of Group III was significantly less than that of Group III (*P* < 0.001).

Though there was no correlation of LF-HF ratio with any of the parameters of Group II, the correlation was significant for WHR, DBP, 30 : 15 ratio and E : I ratio in Group I, and for all the parameters in Groups III and IV (Table 5). However, the degree of correlation was more in Group III compared to that of Group IV, and the magnitude of correlation was more for WHR, DBP, 30 : 15 ratio, and E : I ratio. 

## 4. Discussion

In the present study, the incidence of prehypertension among offspring born to two parents hypertensive and one parent hypertensive was 61.53% (32/52) and 38.46% (20/52), respectively, indicating a higher prevalence of prehypertension in young adults with the parental history of both parents having hypertension. The LF-HF ratio of prehypertensive subjects (Groups III and IV) was significantly higher than that of normotensive subjects (Groups I and II) indicating a considerable sympathovagal imbalance (SVI) in prehypertensives as LF-HF ratio is a marker of sympathovagal balance [21, 25]. This is in corroboration with our recent report that exaggeration of increased sympathetic activity facilitates the onset of hypertension in prehypertensives [27]. However, among the prehypertensives in the present study, SVI was more in offspring of two parents hypertensive as LF-HF ratio of Group III was significantly more (*P* < 0.05) than that of Group IV. 

Till date, no report is available on the HRV analysis and nature of alteration in sympathovagal balance in prehypertensive offspring of hypertensive parents that shifts their autonomic balance towards augmented sympathetic drive. From the present study, it appears that SVI was moderate in prehypertensive offspring of one parent hypertensive and the contribution of sympathetic activation and vagal withdrawal is apparently proportionate for the development of SVI in them. Whereas in prehypertensive offspring of two-parent hypertensive, SVI was more intense and the contribution by vagal withdrawal appears more than the sympathetic activation, as supported by four evidences. First, the general level of significance for changes in HF_nu_ was prominent (*P* = 0.0072) compared to the less prominent changes in LF_nu_ (*P* = 0.0654). As HF_nu_ represents parasympathetic drive and LF_nu_ the sympathetic drive [21, 25], findings of the present study suggest that decrease in vagal drive was more than the increase in sympathetic drive. Moreover, the total power (TP) of HRV spectrum, which represents vagal potency of cardiac modulation was significantly less (*P* < 0.001) in prehypertensives (Table 2) and among them TP was relatively less in offspring of two-parents hypertensive. Second, vagal drive was significantly less in prehypertensive offspring of two parents hypertensive than both the groups of normotensive offspring as HF_nu_ in Group III was significantly less compared to that of Group I and II, whereas changes in LF_nu_ was not significant among the groups (Table 2). Third, significant decrease in RMSSD in Group III compared to that of Groups I and II (Table 3) reflects poor cardiac vagal control in prehypertensive offspring of two parents hypertensive as among the time domain indices of short-term HRV recording, RMSSD specifically reflects vagal modulation of heart rate, and therefore, RMSSD is considered as an important short-term indicator of parasympathetic drive [25]. Thus, significant decrease in RMSSD in Group III prehypertensives reflects poor cardiac vagal control in these subjects. Fourth, the level of increase in basal heart rate (BHR) in prehypertensive offspring (Groups III and IV) compared to their respective normotensive offspring (groups I and II) was significantly higher in group III (*P* < 0.01) than the Group IV (*P* < 0.05) (Table 1). This indicates that the vagal drive in group III subjects was comparatively less; as resting BHR is an index of parasympathetic drive and more the basal heart rate less is the vagal tone [26].

However, LF-HF ratio as assessed by spectral analysis of HRV is not an ideal indicator of sympathovagal imbalance, especially when the heart rate variability is considerably reduced resulting in significant reduction in both LF and HF power. As remarkable decrease in total power of HRV may not be associated with proportionate alterations in LF and HF power, decreased HRV representing poor vagal modulation of cardiac activities could possibly manifest with decreased LF-HF ratio [25]. Therefore, changes in LF-HF ratio should preferably be corroborated with the results of classical autonomic functions tests such as heart rate and blood pressure responses to orthostatic challenges, deep breathing and isometric handgrip.

Heart rate response to standing (30 : 15 ratio, ΔHR_max_, and ΔHR_2min_) and deep breathing (E : I ratio) are parasympathetic function tests, and BP response to standing (ΔSBP_max_, ΔSBP_2min_, ΔDBP_max_, and ΔDBP_2min_) and isometric handgrip (ΔDBP_IHG_) are sympathetic function tests [26]. Significantly high 30 : 15 ratio following standing in Groups III and IV than that of Groups I and II represents decreased vagal activity in prehypertensive offspring compared to that of normotensive offspring, which was further documented by decreased E : I ratio in response to deep breathing in prehypertensive subjects compared to that of normotensive subjects. Moreover, significantly high ΔHR_max_ and ΔHR_2min_ in Group III subjects compared to that of Group IV subjects reflects lower vagal reactivity in prehypertensive offspring of two parents hypertensive than that of one parent hypertensive, as maximum heart rate attained following orthostatic challenge is an inverse indicator of vagal function [26]. 

On standing from supine position, immediately BP falls, which is normally corrected by baroreflex within 15 seconds and there is some degree of overcorrection (ΔSBP_max_ and ΔDBP_max_). Usually, BP returns to a steady state in two minutes, which still remains higher than supine values (ΔSBP_2min_ and ΔDBP_2min_). Changes in SBP reflect change cardiac output, which indicates both vagal and sympathetic reactivities, whereas changes in DBP are due to change in vascular resistance that reflects sympathetic reactivity [26]. In the present study, SBP and DBP changes in response to standing were higher in prehypertensive offspring compared to the normotensive offspring. Also, these responses were more in two-parents hypertensive compared to one parent hypertensive. Thus, these findings reflect increased sympathetic reactivity in prehypertensive subjects, which is more in offspring of two parents hypertensive. This was further confirmed by the presence of higher diastolic response to isometric handgrip (ΔDBP_IHG_) in these subjects, as BP response to handgrip is an important sympathetic function test [26]. 

Thus, the present study reveals that a greater degree of vagal withdrawal and relatively less increase in sympathetic drive contribute to the development of severe SVI in prehypertensive offspring of two parents hypertensive. Though from the present study, the exact cause of sympathovagal imbalance in prehypertensive offspring of hypertensive parents can not be fully ascertained, it could be suggested that adiposity contributes to these autonomic dysfunctions as BMI and WHR in these subjects were significantly correlated with LF-HF ratio (Table 4). Moreover, BMI and WHR of Group III were significantly more than that of Group I and II (Table 1) and the level of significance of LF-HF ratio correlation with these parameters was more in Group III (Table 4). Thus, the degree of adiposity in these high-risk subjects could be a key determinant for the occurrence of prehypertension as obesity has been reported to be either due to or associated with increased sympathetic and decreased parasympathetic activity [28, 29]. Also, the correlation of LF-HF ratio with SBP, DBP, 30 : 15 ratio, and E : I ratio was more prominent in group III prehypertensive subjects. 

The present study reveals that not only the incidence of prehypertension is more prevalent in offspring of two-parents hypertensive, but also they have greater risk of cardiovascular dysfunctions linked to their degree of sympathovagal imbalance and adiposity. The present report is the first of its kind to assess the SVI in young prehypertensive offspring of hypertensive parents. It reveals the importance of vagal inhibition in the possible causation of prehypertension in these young genetically susceptible individuals. Therefore, inspite of limitation of less sample size of prehypertensive offspring, the present study emphasizes the necessity to improve vagal tone and lower sympathetic tone in young offspring of hypertensive parents, especially if both parents are hypertensives so that they do not progress into the stage of prehypertension. Slow breathing exercises have been reported to improve vagal tone and decrease sympathetic tone [30, 31], especially in prehypertensives [32]. Moreover, prior studies by Parati et al. have demonstrated antihypertensive effects of music-guided slow breathing exercises [33, 34]. Therefore, it is proposed that the offspring of hypertensive parents should regularly practice these breathing exercise programs and maintain their sympathovagal homeostasis to prevent the development of prehypertension. 

### 4.1. Limitations of The Study

The major limitation of the present study is the less sample size of prehypertensive groups. The sympathetic drive measured by HRV analysis is not very accurate. Therefore, future studies should include more accurate methods of assessment of sympathetic activity such as estimation of plasma catecholamines or metabolites of catecholamines in urine like vanillylmandelic acid (VMA), metanephrine and normetanephrine. 

## Figures and Tables

**Table 1:** Age, anthropometric, and basal cardiovascular parameters of subjects of various groups.

Parameters	Normotensive offspring		Prehypertensive offspring		*P* values
	Group I	Group II	Group III	Group IV	
	(*n* = 45)	(*n* = 68)	(*n* = 32)	(*n* = 20)	
Age (Years)	20.10 ± 2.80	19.85 ± 2.42	20.05 ± 2.30	19.70 ± 2.21	0.9132
Body weight (Kg)	58.76 ± 5.42	58.45 ± 6.46	62.40 ± 5.84*,^#^	59.10 ± 6.20	0.0199
BMI(Kg/m^2^)	22.70 ± 3.60	22.52 ± 3.45	25.16 ± 4.10*,^##^	24.10 ± 3.40	0.0040
WC (cm)	82.90 ± 6.32	82.28 ± 7.45	86.07 ± 5.56^#^	85.54 ± 5.40	0.0264
WHR	0.840 ± 0.08	0.835 ± 0.09	0.892 ± 0.08*,^##^	0.865 ± 0.07	0.0104
BHR (per min)	70.86 ± 8.70	70.05 ± 8.20	77.50 ± 9.10**,^###^	76.20 ± 8.50^#^	<0.0001
SBP (mmHg)	110.50 ± 7.10	108.65 ± 8.40	128.16 ± 6.20***,^###^	127.30 ± 6.84**,^###^	<0.0001
DBP (mmHg)	72.60 ± 5.90	72.48 ± 6.18	85.96 ± 4.50***,^###^	85.25 ± 4.68***,^###^	<0.0001

Data presented are mean ± SD; Group I: normotensive offspring of both parents hypertensive; Group II: normotensive offspring of one parent hypertensive; Group III: prehypertensive offspring of both parents hypertensive; Group IV: prehypertensive offspring of one parent hypertensive; the *mark indicates comparison with group I, and the ^#^mark indicates comparison with Group II. *P < 0.05; **P < 0.01; ***P < 0.001; ^#^ *P* < 0.05; ^##^ *P* < 0.01; ^###^ *P* < 0.001.

**Table 2:** Frequency domain indices of HRV recorded in supine position of normotensive and prehypertensive offspring of hypertensive parents.

Parameters	Normotensive offspring		Prehypertensive offspring		*P* values
	Group I	Group II	Group III	Group IV	
	(*n* = 45)	(*n* = 68)	(*n* = 32)	(*n* = 20)	
TP (m^2^)	832.50 ± 330.30	882.60 ± 370.40	545.20 ± 210.60***,^###^	605.80 ± 255.70*,^##^	<0.0001
LF_nu_	49.40 ± 18.80	48.12 ± 18.20	58.82 ± 22.70	54.06 ± 20.30	0.0654
HF_nu_	45.35 ± 20.20	46.70 ± 21.26	33.20 ± 14.50*,^##^	38.80 ± 15.40	0.0072
LF : HF ratio	1.90 ± 1.05	1.72 ± 0.95	3.86 ± 1.45***,^###^	3.02 ± 1.15**,^###^,^†^	<0.0001

Data presented are mean ± SD; Group I: normotensive offspring of both parents hypertensive; Group II: normotensive offspring of one parent hypertensive; Group III: prehypertensive offspring of both parents hypertensive; Group IV: prehypertensive offspring of one parent hypertensive; the *mark indicates comparison with group I, the ^#^mark indicates comparison with Group II and the ^†^mark indicates comparison with Group III. *P < 0.05; **P < 0.01;^  ∗∗∗^ *P* < 0.001; ^#^ *P* < 0.05; ^##^ *P* < 0.01; ^###^ *P* < 0.001; ^†^ *P* < 0.05.

**Table 3:** Time domain indices of HRV recorded in supine position of normotensive and prehypertensive offspring of hypertensive parents.

Parameters	Normotensive offspring		Prehypertensive offspring		*P *values
	Group I	Group II	Group III	Group IV	
	(*n* = 45)	(*n* = 68)	(*n* = 32)	(*n* = 20)	
Mean RR (s)	0.845 ± 0.146	0.856 ± 0.151	0.774 ± 0.140^#^	0.786 ± 0.142	0.0313
RMSDD (ms)	26.65 ± 11.30	27.20 ± 12.20	19.80 ± 9.70*,^##^	23.30 ± 10.30	0.0151
NN50	21.70 ± 8.40	22.10 ± 10.10	18.80 ± 7.40	21.90 ± 8.50	0.3663
pNN50	6.02 ± 3.05	6.40 ± 3.10	4.70 ± 2.35	5.65 ± 2.50	0.0546

Data presented are mean ± SD; Group I: normotensive offspring of both parents hypertensive; Group II: normotensive offspring of one parent hypertensive; Group III: prehypertensive offspring of both parents hypertensive; Group IV: prehypertensive offspring of one parent hypertensive; the *mark indicates comparison with Group I, and the ^#^mark indicates comparison with group II. *P < 0.05; ^#^ *P* < 0.05; ^##^ *P* < 0.01.

**Table 4:** Parameters of lying to standing test, deep breathing test, and isometric handgrip test of normotensive and prehypertensive offspring of hypertensive parents.

Parameters	Normotensive offspring		Prehypertensive offspring		*P* values
	Group I	Group II	Group III	Group IV	
	(*n* = 45)	(*n* = 68)	(*n* = 32)	(*n* = 20)	
Lying to standing test					
30 : 15 Ratio	1.20 ± 0.15	1.13 ± 0.19	1.32 ± 0.25*,^###^	1.26 ± 0.20^#^	<0.0001
ΔHR_max_	34.20 ± 6.10	30.80 ± 6.26*	46.50 ± 8.70***,^###^	39.32 ± 5.84*,^###^,^††^	<0.0001
ΔHR_2min_	8.30 ± 2.40	7.20 ± 2.70	12.40 ± 3.10***,^###^	9.82 ± 2.50^##^,^††^	<0.0001
ΔSBP_max_	9.45 ± 2.30	8.34 ± 2.86	13.20 ± 3.10***,^###^	10.32 ± 2.40^##^,^†^	<0.0001
ΔSBP_2min_	5.36 ± 2.42	3.74 ± 1.46***	9.30 ± 2.50***,^###^	6.50 ± 2.08^###^,^†††^	<0.0001
ΔDBP_max_	7.90 ± 2.60	7.24 ± 2.50	10.78 ± 3.50***,^###^	8.20 ± 2.10^##^	<0.0001
ΔDBP_2min_	3.86 ± 2.30	3.60 ± 1.20	6.34 ± 2.20***,^###^	4.80 ± 1.66^†^	<0.0001
Deep breathing test					
E : I Ratio	1.27 ± 0.20	1.35 ± 0.29	1.12 ± 0.16*,^###^	1.19 ± 0.18^#^	<0.0001
Isometric handgrip test					
ΔDBP_IHG_	28.50 ± 5.60	23.40 ± 5.20***	41.10 ± 6.60***,^###^	30.35 ± 5.80***,^###^,^†††^	<0.0001

Data presented are mean ± SD; Group I: normotensive offspring of both parents hypertensive; Group II: normotensive offspring of one parent hypertensive; Group III: prehypertensive offspring of both parents hypertensive; Group IV: prehypertensive offspring of one parent hypertensive; 30 : 15 ratio: ratio of maximum RR interval at 30th beat to minimum RR interval at 15th beat following standing from supine position; ΔHR_max_: maximum rise in heart rate (HR) from supine HR following standing; ΔHR_2min_: maintained increase in HR after attaining steady state at 2 minutes from supine HR level following standing; ΔSBP_max_: maximum rise in systolic blood pressure (SBP) from supine SBP following standing; ΔSBP_2min_: maintained increase in SBP after attaining steady state at 2 minutes from supine SBP level following standing; ΔDBP_max_: maximum rise in diastolic blood pressure (DBP) from supine DBP following standing; ΔDBP_2min_: maintained increase in DBP after attaining steady state at 2 minutes from supine DBP level following standing; E : I Ratio: ratio of maximum RR interval during expiration to minimum RR interval during inspiration following deep breathing; ΔDBP_IHG_: maximum rise in diastolic BP above baseline following sustained handgrip; the *mark indicates comparison with group I, the ^#^mark indicates comparison with group II and the ^†^mark indicates comparison with Group III. *P < 0.05; **P < 0.01; ***P < 0.001; ^#^ *P* < 0.05; ^##^ *P* < 0.01; ^###^ *P* < 0.001; ^†^ *P* < 0.05; ^††^ *P* < 0.01; ^†††^ *P* < 0.001.

**Table 5:** Correlation of LF-HF ratio with BMI, WHR, BHR, blood pressure, 30 : 15 ratio, E : I ratio, and ΔDBP_IHG_ of various groups.

Parameters	Group I		Group II		Group III		Group IV	
	*r*	*P*	*r*	*P*	*r*	*P*	*r*	*P*
BMI	0.018	0.120	0.016	0.140	0.350	0.020	0.312	0.048
WHR	0.310	0.049	0.150	0.110	0.400	0.009	0.380	0.010
BHR	0.285	0.075	0.270	0.080	0.360	0.016	0.354	0.022
SBP	0.280	0.085	0.250	0.090	0.316	0.045	0.312	0.048
DBP	0.320	0.042	0.305	0.050	0.480	0.001	0.400	0.009
30 : 15 Ratio	0.315	0.048	0.276	0.084	0.390	0.006	0.370	0.008
E : I Ratio	0.320	0.047	0.284	0.060	0.485	0.001	0.388	0.004
ΔDBP_IHG_	0.292	0.060	0.220	0.101	0.335	0.025	0.326	0.046

The *P* values less than 0.05 were considered significant.
